# Microbial community patterns in two geochemically contrasting zones within the alkaline lake Bagno dell'Acqua (Pantelleria Island, Italy)

**DOI:** 10.3389/fmicb.2026.1773453

**Published:** 2026-02-27

**Authors:** Agnese Piacentini, Stefano Amalfitano, Barbara Casentini, Andrea Butturini, Ilaria Mazzini, Marco Seminara, Emanuele Colica, Francesco Giuseppe Falese, Francesco Latino Chiocci, Cristina Mazzoni, Stefano Fazi

**Affiliations:** 1 Department of Biology and Biotechnology “C. Darwin”, Sapienza University of Rome, Rome, Italy; 2 CNR-Water Research Institute (IRSA), ARRM1, Rome, Italy; 3 Department of Evolutionary Biology, Ecology and Environmental Sciences, University of Barcelona, Barcelona, Spain; 4 CNR-Institute of Environmental Geology and Geoengineering (IGAG), ARRM1, Rome, Italy; 5 Department of Environmental Biology, Sapienza University of Rome, Rome, Italy; 6 Department of Geosciences, University of Malta, Msida, Malta; 7 CNR-Institute of Environmental Geology and Geoengineering (IGAG), c/o Department of Earth Sciences, Sapienza University of Rome, Rome, Italy; 8 Department of the Earth Sciences, Sapienza University of Rome, Rome, Italy

**Keywords:** alkaline lake, extreme environments, hydrothermal spring, microbial diversity, microbial mat, Pantelleria Island

## Abstract

Alkaline lakes are natural laboratories for studying microbial adaptation to extreme conditions, due to their high pH and often multiple co-occurring stressors. The Lake Bagno dell'Acqua (Pantelleria Island, Italy) is an alkaline crater lake influenced by hydrothermal fluids, which create steep geochemical gradients. We investigated the spatial heterogeneity of the lake's microbial communities and geochemical conditions by sampling both the hydrothermal inflow at different distances from the shore and the water column at the center of the lake in two different seasons (spring, and late summer). Using 16S rRNA gene amplicon sequencing, we characterized the aquatic microbial diversity and inferred the microbial metabolic potential, complementing detailed physicochemical analyses and flow cytometric quantification of microbial abundances. Microbial communities differed markedly between water column and the hydrothermally influenced area along the observed environmental gradient. Water column community was dominated by Actinomycetota, Cyanobacteriota, and Chloroflexota, whereas sediments hosted distinct populations including notable archaeal lineages, such as Thermoplasmatota and Nanoarcheaota. During the spring season, a unique mat dominated by Planctomycetota was observed above the sediment. The area influenced by thermal fluids showed elevated cell abundance and increased taxonomic diversity, consistent with the coexistence of microbial lineages that reflected inputs from both water sources. These findings provide new insights into the distribution and ecological structuring of microbial communities in alkaline–hydrothermal environments and highlights the value of Bagno dell'Acqua as a model for extreme microbial ecosystems.

## 1 Introduction

Alkaline lakes are unique aquatic environments characterized by persistently high pH levels (typically pH > 9), maintained through intense evaporation, the accumulation of dissolved carbonates, and the buffering influence of surrounding carbonate-rich geology (Jones and Grant, 2000; Bozorg-Haddad et al., 2021). As a result, these lakes are retained as open-air laboratories for studying microbial adaptations to extreme conditions and offer a rich source of extremozymes for biotechnological applications (Sorokin et al., 2015).

Microbial life in alkaline lakes thrives in a unique water chemistry and plays a crucial role in biogeochemical cycling, driving transformations in carbon, nitrogen, sulfur, iron, and phosphorus cycles (Haines et al., 2023; Borsodi, 2024; Fazi et al., 2021). One of the main challenges for the aquatic microorganisms is maintaining cellular ion homeostasis. Natronophiles, for example, experience an inverted chemiosmotic gradient, where the external pH is significantly higher than their internal cytoplasmic pH. Sodium (Na^+^) can replace protons (H^+^) as the primary ion driving energy generation and transport processes (Mesbah and Wiegel, 2012; Csitári et al., 2022).

Many alkaline lakes also intersect with geothermal phenomena, which create localized microhabitats with distinct geochemical conditions (Zhang et al., 2023; Karaseva et al., 2024). Hydrothermal springs, fumaroles, and mud pools often discharge into alkaline-saline lakes in volcanic regions. These water inputs typically introduce thermal and geochemical heterogeneity because of hot, ${\text{CO}}_{2}^{-}$rich fluids and gases which alter the temperature, pH, redox state, and mineral content of the receiving water bodies. This environmental heterogeneity is retained as an important variable for high microbial biodiversity (Wobus et al., 2003).

It is now widely accepted that microbial taxonomic and functional diversity processes are driven by a combination of deterministic mechanisms (such as environmental selection) and stochastic factors (such as random dispersal), whose relative influence may vary depending on the type of ecosystem (Stegen et al., 2013; Dini-Andreote et al., 2015). Although these mechanisms have been widely studied in both terrestrial and aquatic environments (Zhou and Ning, 2017; Langenheder and Lindström, 2019), their roles in shaping microbial community structure remain poorly understood in extreme habitats such as alkaline lakes and hydrothermal springs. In particular, the combined influence of multiple co-occurring extreme environmental factors in these systems has yet to be fully elucidated (Barosa et al., 2023; Székely et al., 2013; Dang and Lovell, 2016; Liu et al., 2015).

Understanding the structure and function of microbial assemblages across alkaline–hydrothermal gradients is, therefore, essential to elucidate how geochemical heterogeneity drives ecological differentiation. In such environments, temperature, redox state, and ion composition fluctuate over small spatial scales, creating sharp transitions in microbial diversity and metabolic potential (Podar et al., 2020). Combining microbiological and chemical analyses provides a powerful approach to understand how environmental conditions influence microbial community composition and biogeochemical functioning in these environments, many of which remain poorly characterized from a microbiological perspective.

Lake Bagno dell'Acqua represents one of these environments: an alkaline, hydrothermally influenced lake whose distinct physicochemical features (Mazzoni et al., 2024) and astrobiological relevance (Bruschini et al., 2024; Ubertini et al., 2025) have been documented, but microbial communities are still largely unexplored. Studying microbial communities in this lake offers valuable insights into how life can persist in extreme environments, particularly in relation to pH stress adaptation, temperature fluctuations, and seasonal variability. By assessing microbial abundance and diversity and by estimating microbial functional capability in the alkaline lake Bagno dell'Acqua, this study aims to (1) investigate the spatial geochemical heterogeneity of lake waters and the influence on microbial community structure, (2) compare microbial diversity across two geochemically contrasting zones, and (3) identify microbial “boundary taxa” that may act as ecological connectors between the alkaline lake waters and the more neutral, higher-temperature hydrothermal zone.

## 2 Materials and methods

### 2.1 Study site

The Lake Bagno dell'Acqua is located within the caldera depression of the Caldera Cinque Denti in the northeastern part of the island of Pantelleria. It has a subcircular shape, approximately 450 m long and 350 m wide, with a maximum depth of 12.5 m. The inflow of the lake is determined by direct recharge from precipitation, small tributaries on the western portion of the watershed, as well as inflow from hydrothermal fluid vents rich in CO_2_ primarily located in the southwestern part of the basin (D'Alessandro et al., 1994; Parello et al., 2000; Aiuppa et al., 2007). These hydrothermal fluids contribute to the high alkalinity, salinity, and unique geochemical composition of the lake, fostering microbial processes such as carbonate precipitation and biomineralization (Duchi et al., 1994; Mazzoni et al., 2024). Since the lake has no outlets, its hydrological balance is primarily controlled by meteoric precipitation and evaporation (Aiuppa et al., 2007).

The lake water chemistry is characterized by the following general pattern: Na^+^ > K^+^ > Mg^2+^ > Ca^2+^ for cations and Cl^−^ > ${\text{HCO}}_{3}^{-}$ > ${\text{SO}}_{4}^{2-}$ > ${\text{CO}}_{3}^{2-}$ for anions (Cangemi et al., 2010; Ingrassia et al., 2024). Repeated geochemical surveys have documented the alkalinity of the lake over several decades, with the most recent measurements reporting values of up to 57 meq/L (Jácome Paz et al., 2016).

**Figure 1:**
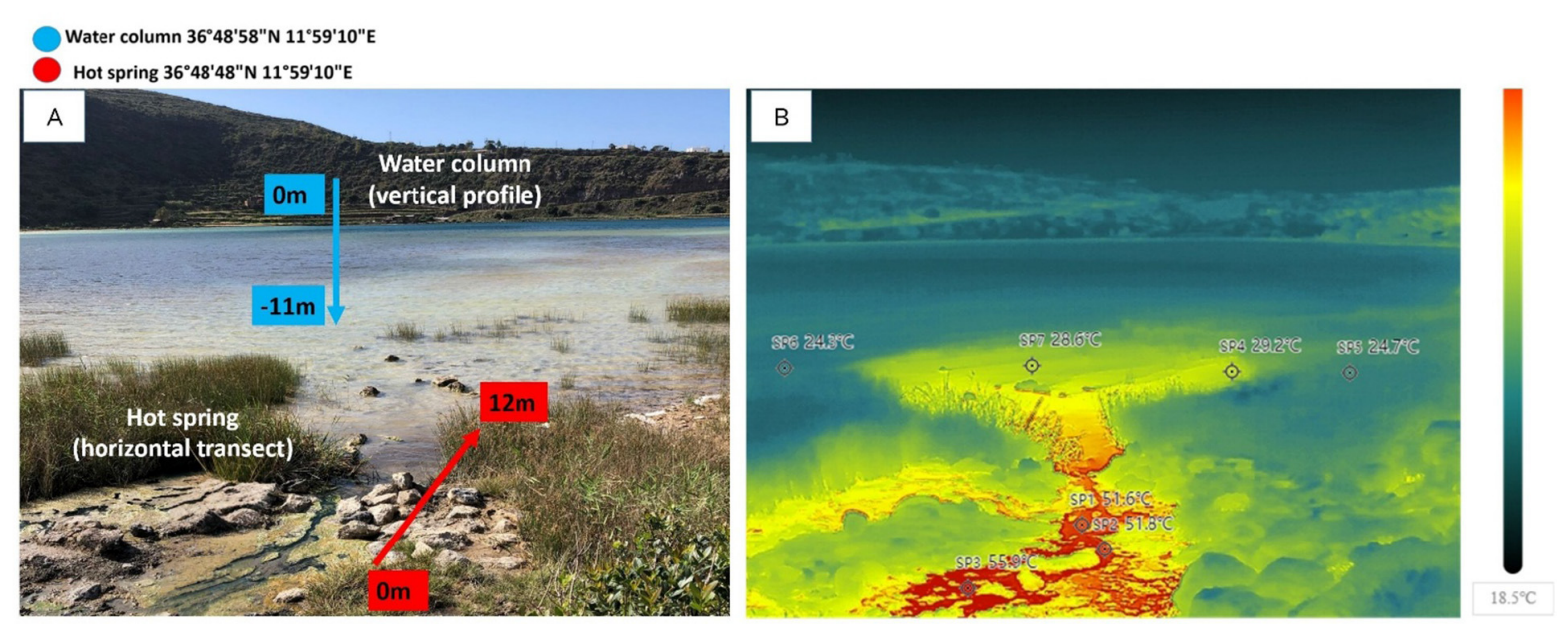
**(A)** Field view indicating the vertical profile of the central lake water column (0,−11 m) and the horizontal transect at the hot spring area (0, 3, and 12 m from the source). **(B)** Infrared thermal image of the hot spring, showing surface temperature gradients (from 18.5 °C to >50 °C) along the transect, taken by drone.

The geological context of Pantelleria Island further influences the lake's chemistry. The island is a Pleistocene stratovolcano, located within a continental rift system between North Africa and Sicily, shaped by tectonic and volcanic processes (Boccaletti et al., 1987). The alkaline nature of the lake results from interactions between hydrothermal fluids and sodium-rich peralkaline rhyolite (pantellerite) (Pecoraino et al., 2015). These processes contribute to its elevated chloride and sodium concentrations, high electrical conductivity (up to 40 mS/cm), and pH values reaching 9 (Pecoraino et al., 2015). The limited water volume makes its chemical composition highly sensitive to hydrological variations, including seasonal precipitation and evaporation cycles. The lake is primarily fed by subsurface springs, which release CO_2_-rich hydrothermal fluids at temperatures between 34 and 58 °C (Cangemi et al., 2018). Thermal springs and bubbling gases are in the southwestern area of the lake (Figures 1A, B).

### 2.2 Sampling procedures

Water temperature (T), pH, and electrical conductivity (EC) were measured using the Hach HQ Series multiparameter probe (Hach Company, Loveland, CO, USA) at regular intervals of 2 m in depth along a vertical profile in the deepest central part of the lake. Moreover, measurements were carried out in correspondence with the inflow of a hot spring along the shore in the southern part of the lake (at 0, 3, 6 and 12 m from the shoreline). All physicochemical measurements were performed *in situ* simultaneously with water sampling during each sampling.

Water sampling at the center of the lake (36°8′58“N, 11°9′13”E) was conducted in spring (25/05/2022) and in September, hereafter named “late summer” (27/09/2022) using a Ruttner bottle along the vertical profile at 0 (CL0m), −2, −3, −5, −7, −9, and −11 m, where negative values indicate depth below the water surface.

Aliquots for nutrient analysis were collected in HDPE bottles and stored at 4 °C until further analysis. For dissolved organic carbon (DOC) analysis, samples were filtered on-site with pre-combusted GF/F Whatman fiberglass filters (Frisenette ApS, Denmark) and acidified with an HCl solution to pH 2 to remove inorganic dissolved carbon and inhibit microbiological carbon degradation. For microbial diversity analysis, lake water (500 mL) was filtered through polycarbonate filters with 0.2 μm pores (GTTP type; diameter 47 mm; Millipore, Eschborn, Germany) and the filters were stored at −20 °C until processing. Unfiltered and filtered aliquots (GFF Whatman) (2 mL) were fixed with a formaldehyde solution (Sigma Aldrich; final concentration 1%) and stored at 4 °C for cytometric analysis.

During the spring sampling, sediment samples and microbial mat (hereafter named ‘'SED1” and “green mat”, respectively) at the bottom were collected by an end-cut syringe operated by divers. Sediment and mat were either directly stored at −20 °C (for the DNA extraction) or fixed with ethanol (for CARD-FISH analysis) (Sigma Aldrich; final concentration 50%) and stored at −20 °C until further processing.

During the late-summer sampling period in addition to the sediment sample collected at the central lake site (SED2), 3 surface water samples were collected at the distances of 0 (HS0m), 3 (HS3m), and 12 m (HS12m) from the shore in correspondence with the inflow of the hot spring of the lake, and respective sediment samples (samples S-HS) were collected and treated as described before.

### 2.3 Characterization of dissolved organic carbon and total nitrogen

Dissolved organic carbon (DOC) and Total Nitrogen (TN) were analyzed with a N/C 3,100 analyzer (Analytik Jena, Germany) (detection limit is 0.1 mg/L). The content of chromophoric and fluoromorphoric organic moieties was estimated with a UV-visible spectrophotometer (UV1,700 Pharma Spec, Shimadzu) and spectrofluorometer (RF-5,301, Shimadzu, Japan) equipped with a xenon lamp and a light-source compensation system S/R mode), respectively.

Water samples were prefiltered with 0.22 μm pore-size PTFE membrane filters (Frisenette ApS, Denmark) to remove impurities, and all optical measurements were performed at room temperature.

Absorbance spectra were recorded in the 200–800 nm range with a 1 cm quartz cuvette. Ultrapure MQ water was used as blank. Two chromophoric descriptors are estimated: the specific aromaticity at 254 nm (SUVA_254_; Weishaar et al., 2003); and the spectral slope at the interval 274–295 nm (S275–295; Helms et al., 2008). SUVA_254_ describes the aromatic content of dissolved organic matter (DOM); S275–295 is typically inversely correlated with the molecular weight of the DOM.

Fluorescence spectra were obtained with a 1 cm quartz cuvette. A three-dimensional excitation-emission matrix (EEM) was obtained for each sample. An EEM consists of 21 synchronous scans with 1 nm increments both in emission and in excitation. The excitation and emission wavelengths ranged from 250 to 410 nm and from 310 to 530, respectively. A 5 nm bandwidth was used for both excitation and emission.

Goletz et al. (2011) protocol was executed to perform the correction and normalization of each EEM. A Raman curve of ultrapure MQ water {λex350 nm, λem371–428nm} (Lawaetz and Stedmon, 2009) was used to normalize. The inner filter effect was removed with absorbance measurements (Larsson et al., 2007).

Three DOM qualitative proxies were estimated with EEMs: the fluorescence index (FI), the freshness index, and the humification index (HIX), following previously published approaches (McKnight et al., 2001; Ohno, 2002; Huguet et al., 2009). Index calculations were performed according to the procedures described by Guarch-Ribot and Butturini (2016). Data are reported as mean ± standard deviation.

### 2.4 Nutrient characterization

Nutrients, namely ammonia, nitrites, nitrates, and phosphate, were spectrophotometrically determined using a UV/VIS spectrophotometer (cell 5 cm, Lambda 25, PerkinElmer, Norwalk, CT, USA). Calibration in fresh and salty water was performed using ${\text{NO}}_{2}^{-}$, ${\text{NO}}_{3}^{-}$ and ${\text{PO}}_{4}^{-3}$ stock solution 1 g/L (Carlo Erba, Italy) and preparing 1 g/L stock solution for ammonia by weighing ammonium chloride (NH_4_Cl) salt (Sigma Aldrich).

In detail, ammonia nitrogen (N-NH_3_) was determined using the Nessler reaction. To 10 mL of sample, 2 drops of Seignette solution were added, followed by 0.4 mL of Nessler reagent. In the case of seawater, a pretreatment step was included to remove interferences from Ca, Na and Mg. Hence, 20 mL of sample were mixed with 200 μL zinc sulfate solution (0.1 g/L ZnSO_4_ · 7H_2_O, Sigma Aldrich) and 150 uL NaOH 6M, prepared by NaOH pellets (Sigma, Aldrich). At higher pH interferents will precipitate and will be removed by centrifugation. 10 mL of surnantant solution will be then treated as above indicated for not saline water. Finally, in both cases (saline and not saline) the colored complex will form after 20 min, and absorption read at 420 nm (APHA, 1998).

Nitrates (N-NO_3_) were determined as nitrites by previously reducing them by passing the solution through a metallic cadmium (Cd) reducing glass column (35 g metallic Cd washed with acetone, 2M HCl and 2% CuSO_4_ solution). The column was finally conditioned with 0.625% NH_4_Cl solution. Then, 100 ml sample mixed with 2 mL 0.625% NH4Cl were passed through the column, first 40 mL discarded and the following 50 mL collected (APHA, 1998). To 10 mL of reduced solution were added of 2 mL of 1% sulfanilamide solution (SA) and 2 mL of 0.1% naphthylethylenediamine solution (NEDA). After a 15 min nitrate read at 543 nm (APHA, 1998). Orthophosphate (${\text{PO}}_{4}^{-3}$) was measured by taking 10 mL sample and adding 300 μL of mixed reagent (45 mL ammonium molybdate solution + 5 mL potassium antimonyl tartrate and 200 mL sulphuric acid 4.5M) then 300 μL ascorbic acid solution. After 20 min absorbance was read at 882 nm (APHA, 1998).

### 2.5 Microbial cell abundance by flow cytometry

The abundance of total prokaryotic communities was determined by the Flow Cytometer A50-micro (Apogee Flow System, Hertfordshire, UK), equipped with a 20-mW solid-state blue laser (488 nm). The light scattering signals, including forward scatter (FSC) and side scatter (SSC), together with red fluorescence (> 610 nm), orange fluorescence (590/35 nm), and blue fluorescence (430–470 nm) were acquired and considered for the direct identification and quantification of distinct microbial groups by following harmonized protocols (Gasol and Morán, 2015). Total prokaryotic communities were quantified by following the staining procedure with SYBR Green I (1:10,000 dilution; Invitrogen™, Thermo Fisher Scientific; Carlsbad, CA, USA; code S7563) Based on the intensity of green fluorescence, prokaryotic cells were further discriminated in high nucleic acid (HNA) and low nucleic acid (LNA). Thresholding was set on the green channel, and the gating strategy was manually adjusted to exclude most of the unspecific signals according to negative unstained controls, consisting of unfiltered samples processed identically but without the addition of SYBR Green I. Thresholding was set on the red channel to exclude most of the unspecific signals according to 0.22-μm filtered control water samples. The gating strategy was manually adjusted on the density plots of SSC vs. Red and of Orange vs. Red channels. The volumetric absolute counting was carried out in density plots of SSC vs. blue channel. Data handling and visualization were performed by the Apogee Histogram Software (v89.0) (Amalfitano et al., 2018).

### 2.6 16S rRNA gene amplicon sequencing

For molecular analyses, water and sediment samples were processed separately. For sediments, 1 g of material was processed per extraction to increase DNA yield. For water samples, 500 mL were filtered at each depth along the central-lake vertical profile (0, −3, −7, −11 m) in both sampling campaigns, and 1,500, 750, and 500 mL were filtered along the hot-spring horizontal transect (0, 3, and 12 m from the source, respectively). Filters were then used directly for DNA extraction. DNA was extracted using the PowerSoil Isolation Kit (MoBio, Carlsbad, CA) according to the manufacturer's instructions. DNA concentration and purity were assessed using a NanoDrop spectrophotometer (Thermo Fisher Scientific, USA), and DNA integrity was verified prior to amplification. A total of 17 samples were sequenced for molecular analyses. Amplicon libraries targeting the archaeal and bacterial 16S rRNA gene (variable regions V4–V8) were prepared using the primer set abeV48A, following a custom amplification protocol. Up to 25 ng of extracted DNA was used as a template for PCR amplification, and each PCR reaction (50 μL) contained 0.5 mM dNTP mix, 0.01 units of Platinum SuperFi DNA Polymerase (Thermo Fisher Scientific, USA), and 500 nM of each forward and reverse primer in the supplied SuperFI Buffer. PCR was done with the following program: Initial denaturation at 98 °C for 3 min, 25 cycles of amplification (98 °C for 30 s, 62 °C for 20 s, 72 °C for 2 min), and a final elongation at 72 °C for 5 min. The forward and reverse primers used included custom 24 nt barcode sequences followed by the sequences targeting abeV48A: [515FB] GTGYCAGCMGCCGCGGTAA and [1391R] GACGGGCGGTGWGTRCA (Apprill et al., 2015; Parada et al., 2016). The resulting amplicon libraries were purified using the standard protocol for CleanNGS SPRI beads (CleanNA, NL) with a bead-to-sample ratio of 3:5. DNA was eluted in 25 μL of nuclease free water (Qiagen, Germany). Sequencing libraries were prepared from the purified amplicon libraries using the SQKLSK114 kit (Oxford Nanopore Technologies, UK) according to manufacturer protocol with the following modifications: 500 ng total DNA was used as input, and CleanNGS SPRI (CleanNA, Waddinxveen, The Netherlands) beads for library cleanup steps. DNA concentration was measured using Qubit dsDNA HS Assay kit (Thermo Fisher Scientific, USA). Fragment size and purity of a subset of amplicon libraries were assessed using an Agilent TapeStation 2,200 automated electrophoresis system with D 1,000 and High Sensitivity D 1,000 ScreenTape (Agilent Technologies, USA). The resulting sequencing library was loaded onto a MinION R10.4.1 flowcell and sequenced using the MinKNOW 22.12.7 software (Oxford Nanopore Technologies, UK).

Reads were basecalled and demultiplexed with MinKNOW guppy g6.4.2 (Oxford Nanopore Technologies, Oxford, UK) using the super accurate basecalling algorithm (config r10.4.1_400bps_sup.cfg) and custom barcodes.

The sequencing reads in the demultiplexed and basecalled fastq files were filtered for length (320–2,000 bp) and quality (phred score > 15) using a local implementation of filtlong v0.2.1 with the settings –min_length 320 –max_length 2,000 –min_mean_q 97. The SILVA 16S/18S rRNA database SSURef NR 99 version 138.1, comprising full-length sequences and formatted using the RESCRIPt pipeline, was downloaded via QIIME 2 on 29 September 2022 (Yilmaz et al., 2014; Robeson et al., 2021). Potential generic place holders and dead-end taxonomic entries were cleared from the taxonomy flat file, i.e., entries containing uncultured, metagenome or unassigned, were replaced with a blank entry. The filtered reads were mapped to the SILVA database SSURef NR99 138.1, which corresponds to a 99% sequence similarity reference, with minimap2 v2.24r1122 (Li, 2018) using the –ax map-ont command and downstream processing using samtools v1.14 (Danecek et al., 2021). Mapping results were filtered such that query sequence length relative to alignment length deviated < 5 %. Noteworthy, low abundant Operational Taxonomic Units (OTUs) making up < 0.01 % of the total mapped reads within each sample were disregarded as a data denoising step. Rarefaction curves were generated to assess sequencing depth and sample coverage, and are reported in supplementary materials (Supplementary Figure 1). Bioinformatic and statistical analyses were conducted in R (v4.2.3) using the ampvis2 (2.7.27) (Albertsen et al., 2015) and iNEXT (2.0.20) (Hsieh et al., 2016) packages for amplicon data analysis and diversity calculations, respectively. The ShortRead (1.54.0) package (Morgan et al., 2009) was employed for quality control and trimming. Lastly, the Seqinr package (4.2.16) was used for the visualization of biological sequences (Charif and Lobry, 2007; Chao et al., 2014). The full OTU abundance dataset generated in this study is available as Supplementary Data 1. Putative ecological functions were inferred using FAPROTAX v.1.2.6 (Louca et al., 2016). The OTU table was reformatted in R v.4.3.2 to obtain a taxonomy-based input, and functional assignments were generated with the FAPROTAX database. The resulting functional matrix was imported in R, where missing values were set to zero, rows with no variance were removed, and data were row-normalized (z-score scaling) prior to visualization as clustered heatmaps with the package pheatmap. The sequencing dataset is available through the Sequence Read Archive (SRA) under accession PRJNA1039605.

### 2.7 Bacterial cell visualization

In order to visualize bacteria within the green mat sample, Catalyzed Reported Deposition-Fluorescence *in situ* Hybridization (CARD-FISH) was applied as described previously (Fazi et al., 2007; Lupini et al., 2011), using a specific rRNA-target Horseradish peroxidase labeled oligonucleotidic probe (PLA46 targeting Planctomycetota, Biomers, Ulm, Germany) (Pizzetti et al., 2011). Cells were then stained with DAPI solution. The stained green mat sample was then observed under a confocal laser scanning microscope (CSLM; ZEISS LSM 900, Carl Zeiss Microscopy GmbH, Jena, Germany) at a magnification of 63 x. Both DAPI-stained cells were excited by 405 nm light and emitted at 430 to 470 nm (blue color). The hybridized bacterial cells were excited with the 488 nm line of an Ar laser (excitation) and observed in the green channel from 500 to 530 nm (emission).

### 2.8 Statistical analysis

Physicochemical parameters were processed and summarized using Microsoft Excel 2016 (Microsoft Corporation, Redmond, WA, USA). The OTU's table was processed in R Studio, and the taxonomic composition was visualized as relative abundances of the most prevalent bacterial and archaeal phyla. Ordination analysis was performed using Principal Coordinates Analysis (PCoA) based on Bray–Curtis dissimilarities (cmdscale, vegan) at the family level. PCoA plots were generated with ggplot2, where the distance between points reflects community dissimilarity, and convex hulls were drawn to highlight sample groupings by habitat and campaign.

Differences in microbial community composition between hot spring water and central lake water were tested using PERMANOVA (adonis2) on Bray–Curtis dissimilarities computed from median-scaled relative OTU abundances.

OTU-level community overlap among sites was visualized using a Venn diagram generated with the R package VennDiagram (Chen, 2022). Unique and shared OTUs were calculated from the non-rarefied OTU table, as sequencing depth was comparable across samples (approximately 80,000 reads per sample).

Observed richness was calculated using the specnumber () function directly from the OTU count table, while Shannon and Simpson diversity indices were computed using the diversity () function implemented in the vegan package (Oksanen et al., 2022). Pielou's evenness was calculated as the ratio between Shannon diversity and the natural logarithm of observed richness. Samples comparisons were visualized with ggplot2 as scatter plots.

## 3 Results

### 3.1 Physicochemical analysis

Lake waters showed an average temperature of 24.2 °C during the spring and of 25.0 °C at the end of the summer with no change along the water depth (Table 1). The direct comparison during the late summer sampling period showed differences between the water column and the hydrothermal spring. In particular, pH and EC remained consistent along the water column, with pH values around 9 and EC around 40 mS/cm. At the hot spring site, the temperature of 52.9 °C decreased with the distance from the source. The pH was more acidic, with values of 6 at the spring, reaching a value of 7.6 at 12 m of distance from the shore. EC values were lower compared to the water column, averaging 15 mS/cm.

**Table 1:** Temperature, pH, and electrical conductivity measured during both sampling, and nutrient concentrations, dissolved organic carbon (DOC), and flow cytometry measurements (total cell count, LNA, and HNA fractions) measured during the late-summer, for water column and hydrothermally influenced samples.

	Spring			Late summer										
	**T (**°**C)**	**pH**	**EC (mS/cm)**	**T (**°**C)**	**pH**	**EC (mS/cm)**	**NH**_3_ **(**μ**g/L)**	${\text{NO}}_{2}^{-}$ **(**μ**g/L)**	${\text{NO}}_{3}^{-}$ **(**μ**g/L)**	${\text{PO}}_{4}^{3-}$ **(**μ**g/L)**	**DOC (mg/L)**	**TCC cells/ml**	**LNA %**	**HNA %**
**Center Lake**														
0m	25.5	9.3	37.5	25.4	9.2	40.8	180.7	< LOD	< LOD	236.0	8.37	6.14E+06	95.5	4.5
−2m	25.5	9.3	37.5	25.1	9.2	40.7	151.5	< LOD	< LOD	831.2	7.37	6.42E+06	90.1	9.9
−3m	25.4	9.3	37.5	25.2	9.2	40.7	413.6	< LOD	6.8	907.5	8.74	6.18E+06	80.6	19.4
−5m	25.2	9.3	37.5	25.2	9.2	40.7	181.5	< LOD	< LOD	536.3	8.27	6.80E+06	83.0	17.0
−7m	23.9	9.3	37.2	25.1	9.2	40.7	105.3	< LOD	< LOD	96.2	7.90	6.07E+06	81.2	18.8
−9m	22.8	9.3	37.0	25.1	9.2	40.6	103.8	< LOD	< LOD	142.0	7.97	6.41E+06	80.2	19.8
−11m	22.0	9.3	36.8	25.1	9.2	40.6	144.7	< LOD	< LOD	190.5	8.08	6.12E+06	78.5	21.5
**Hot Spring**														
0m				52.9	6.4	15.5	339.3	< LOD	355.9	375.0	0.87	3.66E+04	65.5	34.6
3m				42.4	6.9	15.6	305.8	< LOD	154.6	315.1	1.38	2.74E+05	25.5	74.5
6m				42.3	7.1	15.7	83.3	< LOD	158.7	477.1	4.78	4.87E+05	37.7	62.4
12m				39.4	7.6	15.9	221.9	4.6	87.9	214.7	1.42	6.76E+05	45.2	54.8

### 3.2 Dissolved organic matter (DOM) and carbon (DOC) and nutrients

DOC in the water column averaged 8.1 ± 0.43 mg/L without any clear vertical pattern (Table 1). Absorbance spectroscopy revealed little availability of chromophores (SUVA254 averaged 1.56 ± 0.13 L/mg·cm) with the presence of more aromatic and larger DOM molecules (described with the S (275–295) index) at the upper 3 meters. Simultaneously, the fluorescence spectroscopy showed that the fluorescent DOM pool had a low level of humification (HIX < 0.63) with a remarkable signature of tryptophan-like fluorophore at all depths with a maximum at 3–5 m depth, together with the presence of fresh (1.45 < Freshness Index < 2.37) but of moderate microbiological origin (1.3 < FI < 1.36) fluorescent DOM.

DOC at hot spring was much lower (0.87 mg/L) than in the lake and almost free of chromophore substances (SUVA254 = 0.13 L/mg·cm). Moreover, fluorescence spectroscopy indicated that the active hot spring DOM was more humified (HIX = 0.9), older (Freshness Index = 0.92) and with higher contribution of microbiological origin (FI = 1.65) than that reported in the lake water column. As hot water flowed toward the lake, DOC increased up to 4.4 mg/L together with a notable leaching of chromophoric DOM moieties (SUVA254 increases up to 7.2 L/mg·cm). Concurrently, the FI and freshness Index descriptors tended to decrease, and HIX showed a little increase.

Inorganic solutes containing nitrogen and phosphorus showed elevated concentrations in the upper water column (0–5 m), peaking at 3 m depth (Table 1). Concerning nitrogen, ammonia was the dominant species (average 190 μg/L), while nitrites and nitrates were below detection limits in most of samples. In hydrothermal surface waters, nutrient concentrations showed a clear spatial gradient along the horizontal transect (Table 1). At the hot spring, nitrate and ammonium concentrations were highest (356 μg/L and 340 μg/L, respectively) and progressively decreased with distance from the source, reaching 87.9 μg/L of ${\text{NO}}_{3}^{-}$ and 222 μg/L of NH_3_ at 12 m. Phosphate concentrations in hydrothermal waters averaged 354 μg/L and showed no significant spatial trend.

### 3.3 Microbial cell abundance

The total bacterial cell concentration (TCC) in the central lake water varied with depth, reaching 6.8 × 10^6^ cells/ml at−5 m depth (Table 1). In contrast, hot spring water exhibits significantly lower cell concentrations (0.4 – 6.8 x10^5^ cells/ml). Low nucleic acid (LNA) cells dominate the lake samples, particularly at the surface (95.5%), with a progressive decrease at greater depths, while the proportion of high nucleic acid (HNA) cells increased. In the hot spring water, the microbial community showed a higher percentage of HNA cells along the gradient (up to 54.8% at 12 meters).

### 3.4 Prokaryotic community composition

**Figure 2:**
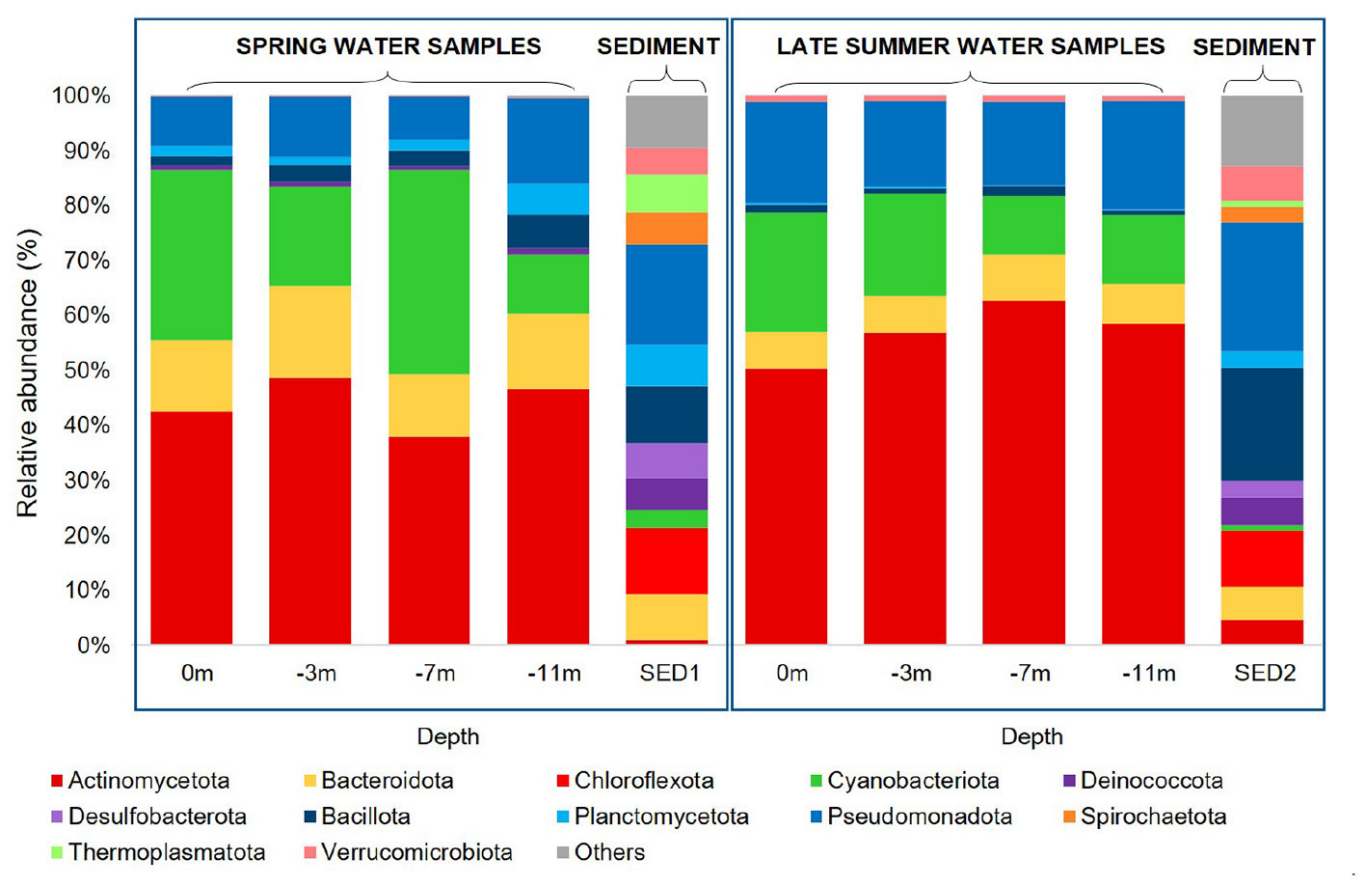
Relative abundance (%) of the most prevalent phyla of Bacteria and Archaea in water samples during spring **(A)** and late summer **(B)** seasons, together with the sediments samples within the two campaigns (SED1 spring, and SED2 late summer). Taxa accounting for ≤ 5% of total reads were grouped into the category “Others”.

Microbial community composition varied with season and habitat, with bacterial phyla dominating all samples, while archaeal phyla were mainly detected in sediments and hydrothermal sites (Figure 2). In the central water column Bacteria dominated with highly similar phylum-level proportions in both seasons— Actinomycetota 50%, Cyanobacteriota 20%, Pseudomonadota 13%, and Bacteroidota 10%—while the spring profile additionally showed minor contributions from Bacillota and Planctomycetota. Actinomycetota were ubiquitous and abundant in both campaigns, with a strong imprint of Micrococcales (31% in spring and 40% in late summer sampling period) and a secondary contribution of Microtrichales detectable only in late summer (3%) (Figures 2A, B). Among Cyanobacteriota, Synechococcales dominated (24% in spring vs. 16% in late summer) but decreased at −11 m (10%), where Pirellulales, absent along the column, rose to 6%, consistent with the planctomycetal mat recorded at the water-sediment interface during the late summer sampling campaign. Burkholderiales increased in late summer waters (9% vs. 3%), while Archaea were not detected in the water column in both seasons.

In the sediments of the central lake (SED1 spring; SED2 late summer), phylum-level composition was broadly conserved across seasons, dominated by heterotrophic and anaerobic lineages: Pseudomonadota 18–22% (mainly Gammaproteobacteria, families *Defluviicoccaceae* and *Ectothiorhodospiraceae*, along with a fraction of Alphaproteobacteria), Bacillota 10–20% (classes Bacilli, Dethiobacteria; families *Acholeplasmataceae, Dethiobacteraceae*), Chloroflexota 10–12% (Anaerolineae, Dehalococcoidia), and Bacteroidota 6–9% (Bacteroidia, Rhodothermia). In sharp contrast to the water column, sediments hosted a substantial and seasonally modulated archaeal fraction. During the spring season, the archaeal component was higher than in late summer and dominated by Thermoplasmatota. In particular, Thermoplasmata accounted for 72.7% of archaeal reads during the spring season and 68.4% in late summer. Nanoarchaeota were the second most abundant group, represented by the class Nanoarchaeia (14% in spring, and 10% in late summer) followed by Micrarchaeota (6 % spring, and 2 % in late summer). Crenarchaeota, represented by the class Bathyarchaeia, were detected exclusively in spring samples (4%).

**Figure 3:**
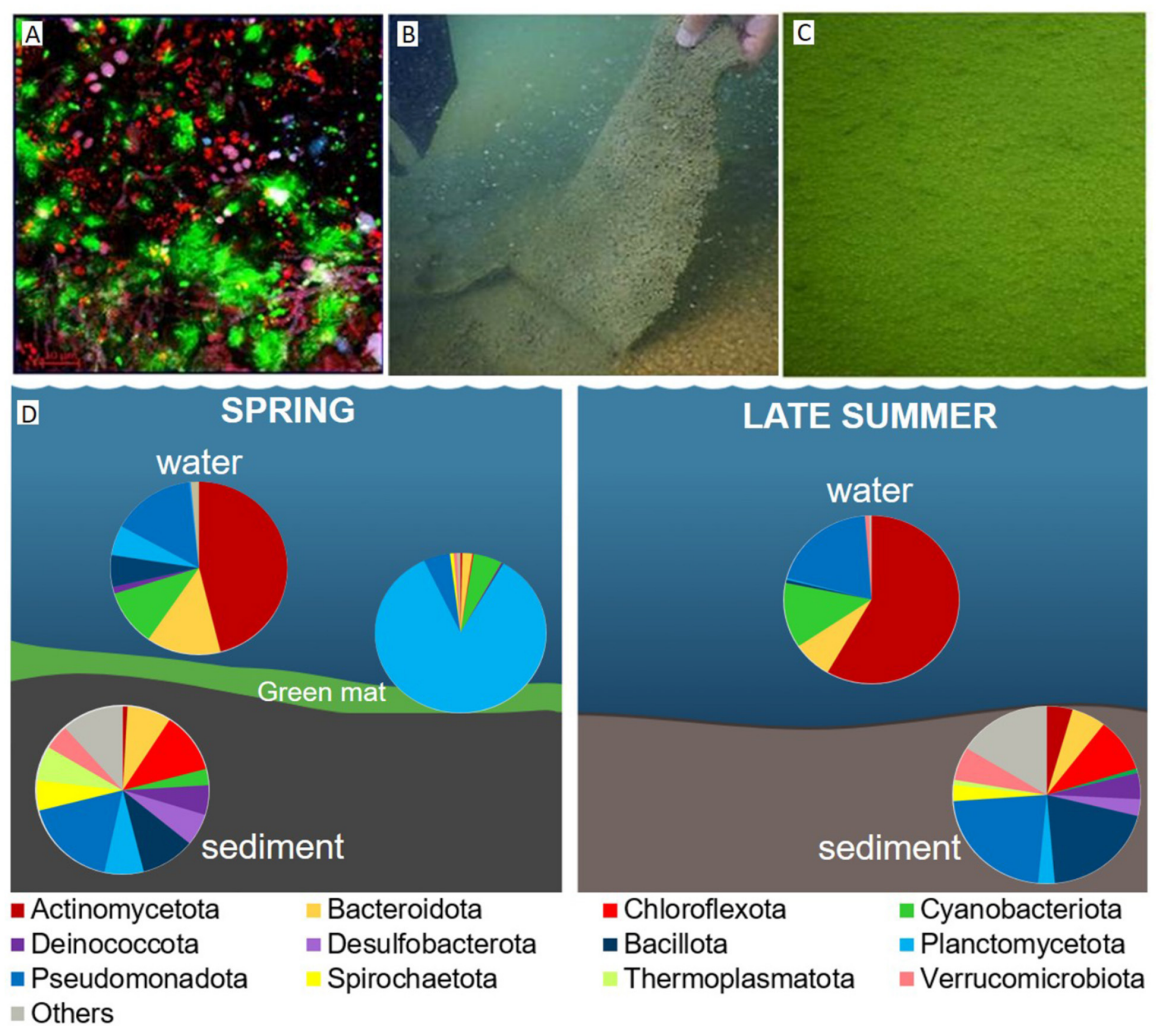
**(A)** CLSM combined images showing the spatial distribution of the Plantomycetota phylum (green) and other DAPI stained cells (blue) identified by CARD-FISH in the *green mat* sample. Autofluorescent cells appear in red. The hybridized bacterial cells were excited with the 488 nm line of an Ar laser (excitation) and observed in the green channel from 500 to 530 nm (emission). Bar = 20 μm. **(B, C)** Images of the sample ‘'*green mat”* taken at a depth of−11 meters during the spring campaign (photos by Ilaria Mazzini and Giovanni Gaglianone). **(D)** Taxonomic composition of microbial communities in the water at the bottom of the lake (-11m), in the green mat, and underlying sediments during spring (left) and late summer (right).

The green mat (Figure 3), present only during the spring season, physically separated the oxygenated deep water from the underlying anoxic sediment. Its community was dominated by Planctomycetota, as shown in Figures 3A, 3D (85%; families *Pirellulaceae, Rubinisphaeraceae*), while Cyanobacteriota (5.5%, mainly *Cyanobiaceae*) and Pseudomonadota (5%, families *Rhodobacteraceae, Rhizobiaceae*), occurred at lower relative abundances. The planctomycetal signature recurred both in the deep water at −11 m and in the overlying sediment (Figure 3D). No Archaea were detected in this sample.

**Figure 4:**
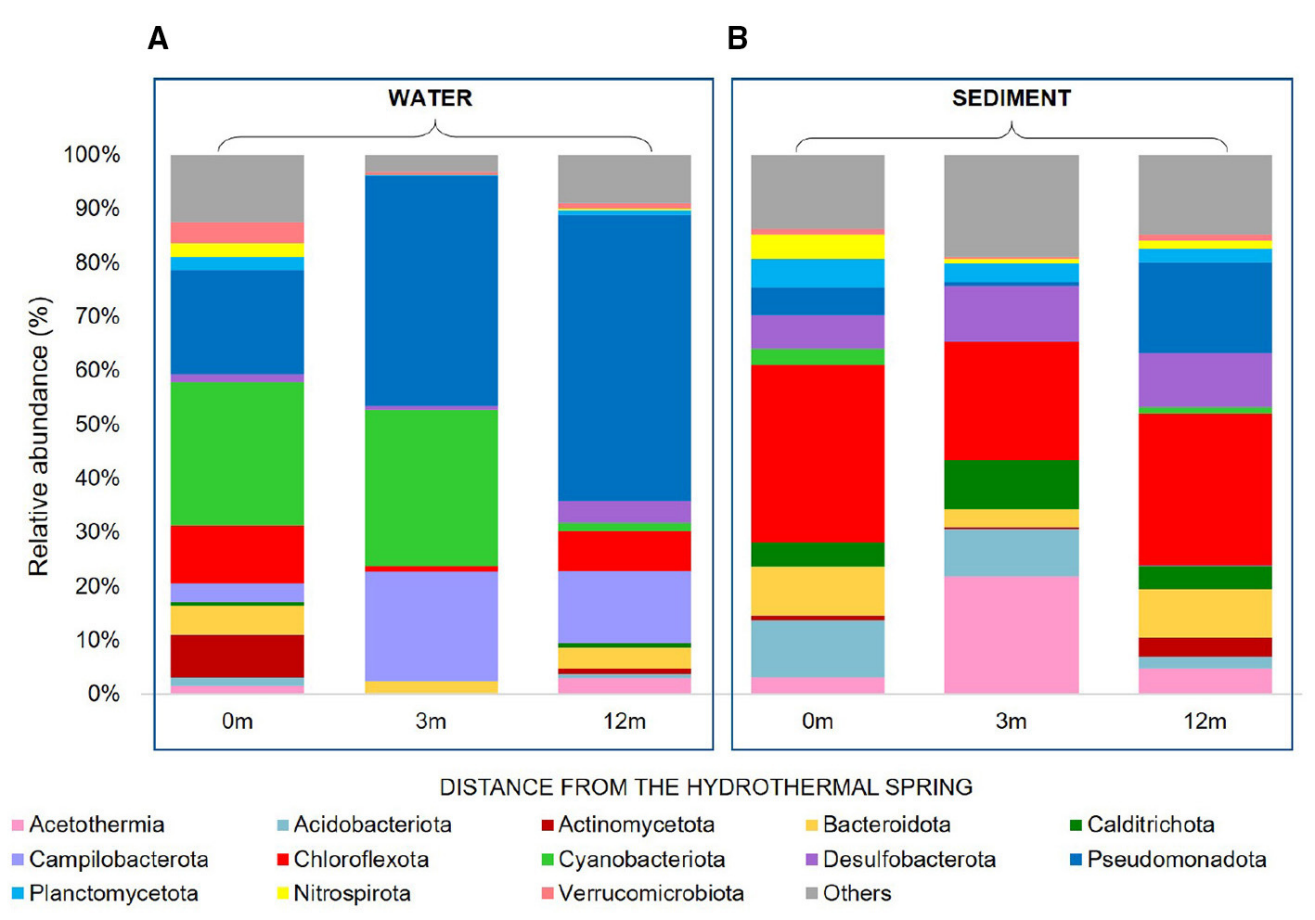
**(A)** Relative abundance (%) of the most prevalent phyla of Bacteria and Archaea in water and **(B)** sediments samples taken during the late summer season at different distances from the hydrothermal spring (0 m, 3 m, 12m). Taxa accounting for ≤ 5% of total reads were grouped into the category “Others”.

In the hot spring samples (Figure 4), the horizontal transect revealed a strong water–sediment disjunction and pronounced spatial structuring. In the hot spring waters (Figure 4A), Cyanobacteriota (Cyanobacteria class, including *Cyanobium* and *Synechococcus* genus) and Pseudomonadota (mainly Gammaproteobacteria, including Pseudomonadales, Burkholderiales) dominated, with gradual changes along the transect. Cyanobacteria class was abundant in water near the vent (27% at 0 m) but declined sharply in sediments (3%). In the hot spring sediments (Figure 4B), taxa typical of reduced niches prevailed, including Chloroflexota (Anaerolineae), Desulfobacterota, and Acidobacteriota. At 3 m from the vent, sediments showed a marked peak of Acetothermia (22.4%), absent in the co-localized water. At 12 m, the water–sediment divergence was even stronger, with Chloroflexota reaching 29.6% in sediments vs. 7.6% in water, while Pseudomonadota accounted for 55% of the water but only 17.7% of the sediment.

The archaeal component in hot spring samples also followed distinct gradients. At 3 m in the water, communities were dominated by Nanoarchaeota (Nanoarchaeia, 41.3%), together with Iainarchaeota (25.8%) and Halobacterota (13.7%; classes Methanosarcinia, Methanomicrobia). In contrast, Crenarchaeota (38.7% Bathyarchaeia*)* and Thermoplasmatota (33% Thermoplasmata) dominated 3 m sediments. At 12 m from the spring, water sample showed the highest contribution of Halobacterota (56%), co-occurring with Nanoarchaeota (40%) and a smaller fraction of Altiarchaeota (4%, exclusive to water).

**Figure 5:**
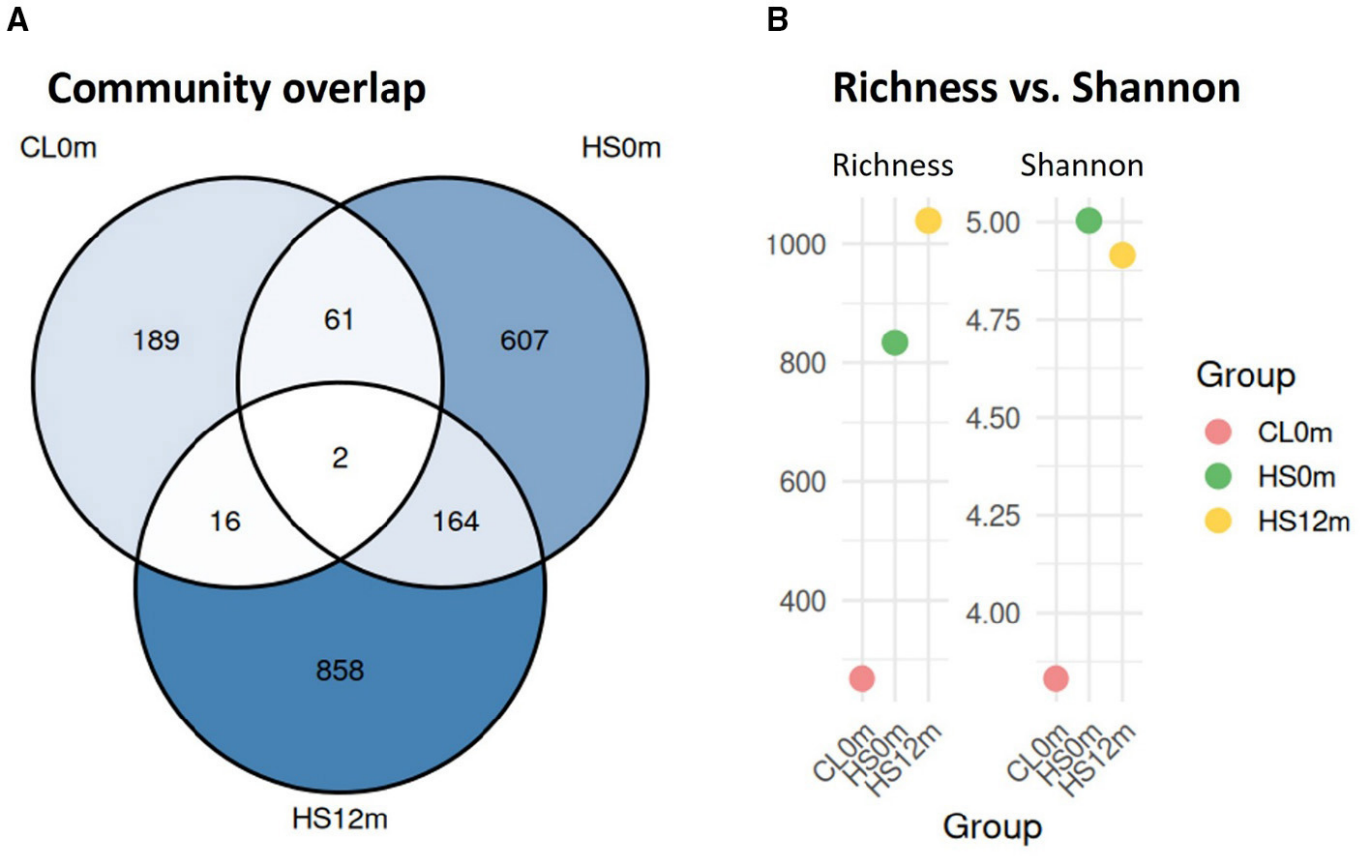
**(A)** Overlap of OTUs among surface-water communities from the central lake (CL0m), the hydrothermal spring (HS0m), and a site 12 m from the spring (HS12m). **(B)**. Richness and Alpha diversity metrics from CL0m to HS0m to HS12m, with HS12m samples showing the highest richness in 12m from the hydrothermal spring.

Community overlapped at the OTU level (Figure 5A) showed pronounced spatial structuring across the three surface sites. Only 2 OTUs were shared by the three habitats (*Roiseinatrobacter sp*. and *Rhodobacteraceae_bacterium*), whereas most OTUs were unique to HS12m (858; 45%), followed by the hydrothermal vent (607; 32%) and the central lake surface water (189; 10%). Alpha diversity metrics varied across samples along the surface transect spanning the hydrothermally influenced area and the central lake water (Table 2). Observed richness was lowest in the central lake surface water (CL0m), increased markedly at the hot spring (HS0m), and reached the highest value at 12 m from the hydrothermal spring (HS12m). Shannon diversity showed a different pattern, with the highest value observed at HS0m (Shannon = 5.00), followed by HS12m (Shannon = 4.91), and lower diversity in the central lake surface water (CL0m; Shannon = 3.83). Similarly, Simpson diversity and Pielou's evenness were highest at HS0m, with a more even distribution of taxa at the hot spring compared to the other sites.

**Table 2:** Alpha diversity indices (observed richness, Shannon diversity, Simpson diversity, and Pielou's evenness) calculated for individual surface water samples collected along the hydrothermally influenced transect, including the central lake surface water (CL0m), the hot spring (HS0m), and the site located 12 m from the hot spring spring (HS12m).

**Sample**	**Richness**	**Shannon**	**Simpson**	**Pielou**
CL0m	268	3.831.897	0.945	0.685
HS0m	834	5.003.324	0.968	0.743
HS12m	1040	4.913.528	0.929	0.707

**Figure 6:**
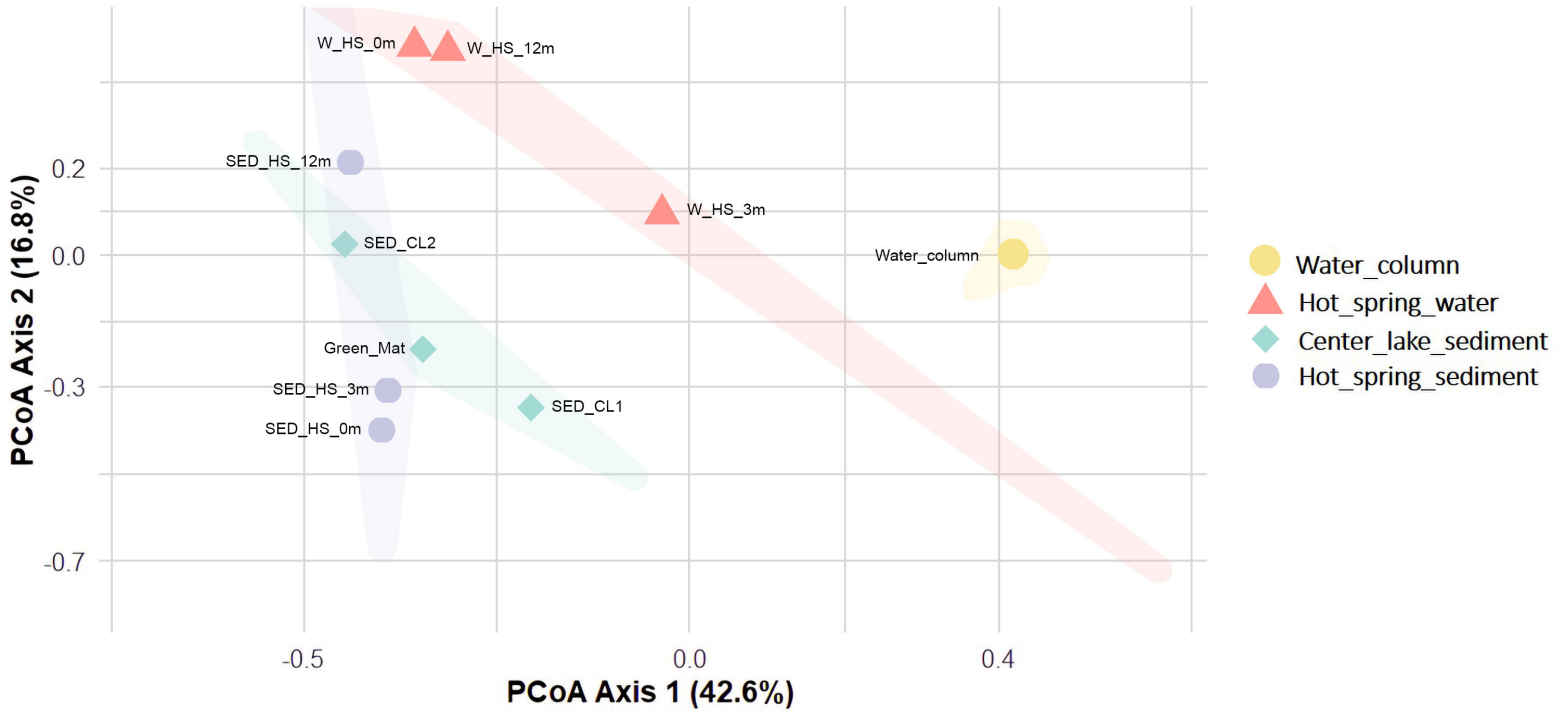
Principal Coordinates Analysis (PCoA) of microbial community composition based on Bray–Curtis dissimilarities at the family level. Each point represents a single sample; the distance between points reflects the degree of community dissimilarity. Samples are grouped by matrix type. Ellipses indicate the 90% confidence region of the sample distribution based on the first two PCoA axes.

Beta-diversity analysis (PCoA based on family level) further highlighted differences in microbial community composition among habitats (Figure 6). Axis 1 (42.6% variance) clearly separated hot spring waters from all other samples, while Axis 2 (16.8% variance) distinguished hot spring sediments, central lake sediments, the green mat, and water column. Pairwise PERMANOVA analyses revealed significant differences in microbial community composition at the family level between water column and central lake sediment (*R*^2^ = 0.65, p_adj = 0.014), and between water column and hot spring water (*R*^2^ = 0.59, p_adj = 0.014), with no significant differences in multivariate dispersion between groups (*p* = 0.07).

**Figure 7:**
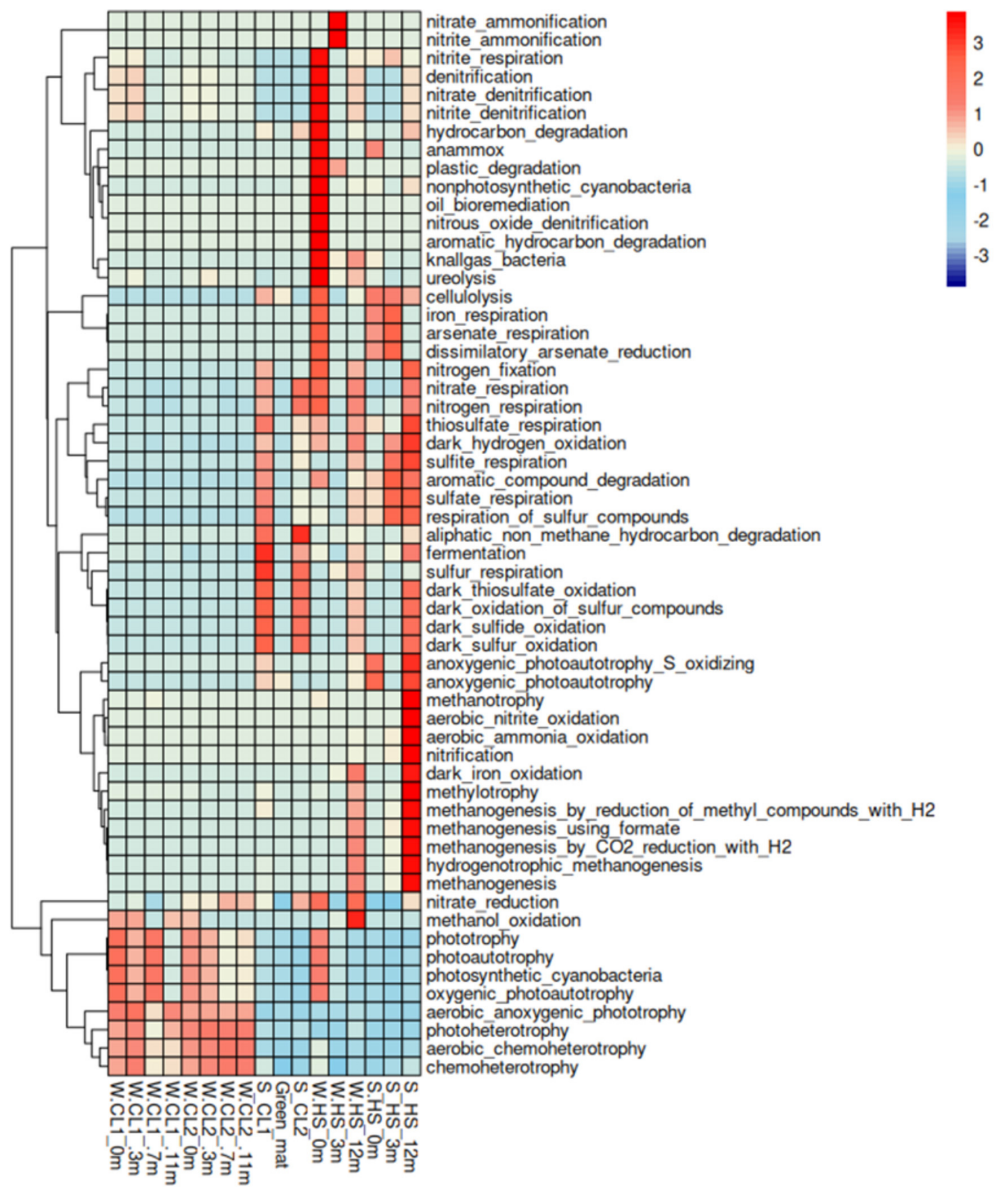
Distribution of metabolic functions obtained from FAPROTAX. The matrix was row-normalized using Z-score standardization to highlight relative variations in each metabolic function across samples. Columns represent the samples, while rows correspond to the annotated metabolic functions. The color gradient ranges from blue (low relative abundance) to red (high relative abundance).

FAPROTAX predictions indicated an enrichment of sulfur cycling pathways, methanogenesis, and hydrocarbon degradation in hot spring sediments (Figure 7). In contrast, the central lake water column, both in spring and late summer, was enriched in functions associated with phototrophy, chemoheterotrophy, and nitrogen cycling (nitrification and nitrite respiration). Central basin sediments showed higher contributions of fermentation, anaerobic respiration, and aromatic compound degradation, with the spring samples displaying a stronger representation of functions linked to anoxic processes compared to the late summer samples.

## 4 Discussion

The Lake Bagno dell'Acqua, with its moderate alkaline and saline character combined with hydrothermal input, represents an extreme ecosystem where sharp physicochemical gradients with chemical analyses reveal a pronounced geochemical contrast between the main lacustrine water mass and the hydrothermal spring. The temperature profile was relatively homogeneous, indicating active mixing during both seasons. The lack of detectable nitrate, together with the presence of ammonia, indicated that nitrogen cycling shifted toward reduced forms. Under the lake's elevated pH, the ammonium–ammonia equilibrium is displaced toward un-ionized NH_3_, which may contribute to nitrogen loss while simultaneously inhibiting nitrification (Amatya et al., 2011).

The hot spring introduces pronounced nutrient gradients. At 0 m from the source, elevated nitrate concentrations (356 μg/L) were observed alongside high ammonium levels (340 μg/L ). As the distance from the source increases (up to 12 m), nitrate concentrations dropped sharply (87.9 μg/L), and the pH gradually returns toward neutrality. This suggests that the thermal water delivers an input of oxidants (e.g., nitrate) and reduced substrates (NH4^+^) into an environment otherwise depleted in oxidized nitrogen forms (Liang et al., 2024).

Hypersaline alkaline lakes consistently exhibit a reduced efficiency of the nitrogen cycle (accumulation of ammonium, incomplete nitrification) and high primary productivity driven by alkaliphilic cyanobacteria (Sorokin et al., 2015). The rapid disappearance of nitrates with increasing distance suggests biological assimilation or local denitrification, favored by reducing conditions in the sediments adjacent to the spring (Dodsworth et al., 2011; Feng et al., 2023). At the same time, phosphate concentrations near the spring remain in a constant range (350–470 μg/L), reflecting a geothermal contribution and possibly release from underlying sediments. The highest values of ${\text{PO}}_{4}^{-3}$ between 3–5 m in the water column, correspond to the higher value also of fluorescent organic carbon signature, suggesting that degradation activity carried out by bacteria (TCC also had its maximum at 5 m depth) together with other metabolic patterns may contribute to this concentration trend. In addition, the co-occurrence of inorganic nitrogen and phosphorus species and fluorescent organic matter in the 3–5 m layer may also be associated with the presence of algal cells, which typically bloom at this depth (Yaakob et al., 2021).

In alkaline lakes, dissolved phosphate often reaches unusually high concentrations, as both geochemical precipitation and biological uptake are reduced under high pH conditions (Diaz et al., 1994; Toner and Catling, 2020; Haas et al., 2025). However, in Bagno dell'Acqua phosphate concentrations were comparatively lower than those typically observed in extreme soda systems. Previous studies at this site have shown that phosphate can non-etheless be immobilized under alkaline, ion-rich conditions, and that microbially mediated processes may contribute to this removal. In particular, bacterial isolates from Bagno dell'Acqua were shown to precipitate hazenite, a phosphate mineral, under laboratory conditions (Mazzoni et al., 2024). Such biologically driven mineral precipitation offers a plausible mechanism explaining why dissolved phosphate remains low in the lake despite its alkaline chemistry (Pecoraino et al., 2015) and the presence of sodium-rich waters, which can enhance phosphate solubility under certain conditions (Grant, 2006).

Overall, geochemical and hydrothermal studies of Pantelleria Island and the Lake Bagno dell'Acqua (D'Alessandro et al., 1994; Pecoraino et al., 2015; Cangemi et al., 2018) characterize the system as an extreme environment, with an oxidizing, alkaline water mass enriched in carbonates and localized geothermal inputs that generate warmer, lower-pH, and more reducing microenvironments. Such physicochemical gradients act as major drivers in shaping microbial community structure, providing distinct energy and nutrient niches within the same ecosystem (He et al., 2021; Pardo-Esté et al., 2023; George et al., 2023).

The environmental gradients were also reflected in the flow cytometry data. In the water column, the total concentration of prokaryotic cells (average TCC = 6.31 × 106 ± 2.59 × 105 cells/mL) remained relatively constant with depth, with differences in percentage of HNA vs. LNA cells. The deeper lake layers harbored a higher proportion of HNA cells, whereas surface waters were dominated by LNA cells, which are considered more quiescent (Song et al., 2019). This pattern was reported to be related to the greater availability of organic substrates near the sediment and differences in oxygen concentration (Morán et al., 2007).

The pronounced shift in microbial abundance and community structure observed along the hydrothermal gradient indicated that the zone between spring inflow and lacustrine waters created ecological conditions favorable to microbial proliferation. In this transition area, thermophilic stress-tolerant taxa are progressively replaced by more metabolically active populations, a pattern consistent with microbial successions described in other hydrothermal systems (He et al., 2021; Karaseva et al., 2024).

Overall, the initial thermophilic pioneers were followed by more metabolically active communities as environmental conditions became more favorable. This pattern aligns with the concept of “cascading ecosystems” in hydrothermal springs, where decreasing physical stress along the outflow path enables exponential increases in biomass and biological activity (Hu et al., 2023).

DOC concentration (8.1 ± 0.4 mg/L) indicated a moderate amount of available organic substrate in the water column, with no pronounced vertical gradients. However, these concentrations are not as high as those reported in other soda lakes or in shallow hypersaline lakes (Butturini et al., 2020, 2022). Fluorescence data further point to a low degree of humification throughout the profile (HIX < 0.63), which is typical of labile organic matter. In contrast to those systems, SUVA values were low, suggesting that dissolved organic matter was not likely to interfere with solar radiation (Helms et al., 2008). This allows light to penetrate deeply through the water column and potentially support photosynthesis even in central deep areas. Such conditions are consistent with a productive lake system (Feng et al., 2025), where organic carbon is primarily of autochthonous and recent origin rather than derived from humified terrestrial matter. In line with this, many alkaline-saline lakes show high primary production due to the abundance of photosynthetic cyanobacteria, as also detected in this study through 16S rRNA gene analyses, resulting in a continuous supply of DOC (Fazi et al., 2021; Gao et al., 2022). In Lake Bagno dell'Acqua, the significant presence of planktonic cyanobacteria supports this interpretation. The combination of bioavailable DOC and non-limiting nutrient concentrations fuels intense microbial metabolic activity within the system. This is indirectly evidenced by the accumulation of ammonium (a final product of remineralization) and the high proportion of HNA cells.

The microbial community of the lake showed a compositional diversity strongly influenced by both habitat compartment (pelagic water vs. benthic sediment) and sampling period. Overall, the microbial assemblage is dominated by a few phyla adapted to alkaline-saline conditions, belonging mainly to the phyla Actinomycetota, Bacteroidota and Pseudomonadota (Bell et al., 2018; Bullerjahn et al., 2020; Szabó et al., 2017). In the open water, Actinomycetota (particularly Actinobacteria, family *Nitriliruptoraceae*) constitute the most abundant clade (45–50% of sequences), as also reported for other alkaline environments (Bowers et al., 2009). Cyanobacteriota represented the second most abundant group (20–25%), mainly consisting of *Cyanobium* and *Synechococcus*. Pseudomonadota (especially Gamma- and Alphaproteobacteria) and Bacteroidota also contribute modestly to the planktonic community as reported in Van soda lake (Ersoy Omeroglu et al., 2021).

In contrast, sediments hosted more heterogeneous communities typical of environments with low oxygen concentrations. Anaerobic Chloroflexota (class Anaerolineae, 28% in sediment near the hydrothermal spring), along with Bacillota and Desulfobacterota, emerged as abundant groups. Actinomycetota and Pseudomonadota were present across both compartments. Members of the genus *Defluviicoccus*, which are capable of glycogen accumulation (Burow et al., 2007), could contribute to the anaerobic biodegradation of organic material in the sediment. Such ecological stratification between oxygenated water and reduced sediment is common in extreme saline lakes and results in a clear differentiation of microbial communities between the two compartments (Antony et al., 2013).

Alpha diversity metrics, specifically the Shannon index, reflected the differences between sediment and open-water microbial communities. Sediment samples exhibited higher diversity values and more even species distributions compared to open waters, where a few phylogenetic lineages dominate (Sauer et al., 2022). Sequencing data show that sediments contained numerous additional low-abundance phyla (Bacillota, Desulfobacterota, Planctomycetota, Nanoarchaeota, and other Archaea) that contribute to the overall microbial richness. In contrast, pelagic communities, dominated by photosynthetic taxa, showed lower Shannon index values as observed in previous studies (Zhang et al., 2020; Vettorazzo et al., 2024).

Sampling conducted in two different sampling periods highlighted changes in microbial community composition, suggesting a marked seasonal dynamic. During spring, in particular, a conspicuous green phototrophic microbial mat (“green mat”) was observed at the lake bottom (−11 m), forming a continuous layer across the entire water–sediment interface, while this mat was not present during the autumn season. A similar seasonal dynamic has been reported in Hot Lake (Washington, USA), which harbors a benthic phototrophic mat that assembles each spring and disassembles each fall, reflecting strong seasonal patterns in microbial mat development linked to light availability and nutrient inputs (Lindemann et al., 2013). Taxonomic analysis of the green mat revealed a community almost entirely dominated by Planctomycetota, with minor contributions from Cyanobacteriota and Pseudomonadota. Planctomycetota is an enigmatic bacterial phylum that follows complex lifestyles and display unusual cell biological features. Members of Planctomycetota are generally known for having large genomes (Andrei et al., 2019; Glöckner et al., 2003), which might indicate large phenotypic plasticity and the ability to quickly adapt to extreme changes in their environment (Storesund et al., 2020). Their physiology is particularly geared toward the degradation of polysaccharides derived from phototrophic organisms (Kündgen et al., 2025). During the same season, planctomycetal lineages were also detected in the deep water at −11 m and in the sediment beneath the mat, suggesting a continuity between the mat and adjacent habitats. Moreover, it was demonstrated that anaerobic metabolic capabilities are widespread across major Planctomycetota lineages, with carbohydrate fermentation and sulfur reduction likely supporting their survival under anoxic conditions (Elshahed et al., 2007; Shao et al., 2023). The presence of the green microbial mat at the bottom showed that light was likely to penetrate the entire water column facilitated by the low humic substances content (i.e., low SUVA values). However, we hypothesize that the phototrophic organisms within the mat release oxygen into the overlying water. This could promote oxic conditions at the sediment–water interface, favoring the establishment of diverse microbial assemblages. By contrast, the absence of this mat during the late summer allowed greater interaction between the sediment and the overlying waters; the community composition was correspondingly less enriched in Archaea.

The observed strong seasonality in the dynamics of this mat is in line with previous studies where microbial mats are typically reported to disintegrate in winter and proliferate under favorable irradiance and nutrient conditions (Bolhuis and Stal, 2011; Cardoso et al., 2019).

PERMANOVA revealed significant differences in microbial community composition at the family level between hot spring water and central lake water. The source of the thermal spring water was rich in cyanobacteria with 27% of sequences assigned to class Cyanobacteria, as reported in previous studies (Keshari et al., 2022; Lindemann et al., 2013) and in Chloroflexota with the family *Anaerolineaceae*, whereas the underlying sediments showed markedly higher taxonomic diversity, and phototrophic cyanobacteria drop below 3%. With increasing distance (3 m, and 12 m) in the water samples, there was a marked enrichment in Pseudomonadota (families *Thiomicrospiraceae, Halothiobacillaceae*, and *Ectothiorhodospiraceae*) and in the phylum Campylobacterota (families *Arcobacteraceae, Helicobacteraceae*, and *Sulfurimonadaceae*). At 12m of distance, the community was characterized by a distinct taxonomic composition with different taxa from both the thermal environment (e.g., moderate thermophiles, archaeal Thermoplasmatales, chemoautotrophic bacteria) and the alkaline lake (e.g., halophiles, cyanobacteria), creating a mixed community with high local diversity. In this spot, the microbial community displayed the highest richness, indicating a greater number of OTUs compared to both the surface central lake and the spring source waters (Figure 5). However, the Shannon index was highest in the spring source, possibly reflecting greater evenness in the relative abundance of taxa at the source (Kumar et al., 2024).

The emerging functional analysis reflected the environmental gradients, the open waters of the lake are dominated by photoautotrophic functions (oxygenic photosynthesis by Cyanobacteriota) and aerobic heterotrophy (organic matter degradation by Actinomycetota and Bacteroidota), in line with an oxygenated, illuminated, and DOC-rich environment (Xie et al., 2024; Borsodi, 2024; Feng et al., 2025). Furthermore, probably part of the ammonia undergoes to autothropic oxidation due to the presence of ammonia-oxiding bacteria as reported in other hydrothermal marine contexts (Lam et al., 2004). In the sediments, predicted functions shift toward anaerobic metabolism, so processes such as fermentation, anaerobic respiration (e.g., sulfate or alternative electron acceptor reduction) and methanogenesis predominate. The functional profiles of central lake sediments displayed marked seasonal differences, likely influenced by the presence of the green mat. In particular, spring sediments were enriched in metabolic pathways associated with anoxic processes, including nitrogen fixation, nitrate and thiosulfate respiration, sulfite and sulfate respiration, and the degradation of aromatic and aliphatic hydrocarbons. These patterns confirmed that microbial activity in spring is strongly shaped by the development of the green mat, which creates localized anoxic niches that favor the diversification of anaerobic metabolisms, which reflect the presence of anaerobic Chloroflexota, sulfate-reducing bacteria, and methanogenic archaea within the benthic communities (Rojas et al., 2018).

A remarkable result emerging from the functional analysis is the drastic change observed in the area located 12 m from the hydrothermal source as a metabolic hotspot, where richness exceeded that of both the spring and lake waters under more neutral temperature and pH conditions. This aligns with the hypothesis that polyextreme habitats limit the growth of many microbial taxa, a trend reported in both geothermal and non-geothermal environments alike (Lauber et al., 2009; Sharp et al., 2014; Power et al., 2018). Water and sediment samples collected at 12 m from the thermal spring exhibited the broadest spectrum of predicted metabolic functions and some of the highest relative abundances for many pathways, according to the FAPROTAX analysis. The potential transitional zone where geothermal water [(39 °C9 °C, pH 7.6)] mixes with the alkaline lake water supports diverse microbial assemblages potentially associated with multiple biogeochemical pathways. In this zone (HS12m) a continuous input of nutrients (N and P) and electron donors from the geothermal flux support a variety of metabolic processes. The aquatic community included chemolithotrophic lineages such as nitrifiers and sulfur oxidizers, thriving on steep redox and chemical gradients, together with phototrophic and heterotrophic taxa, indicating the coexistence of diverse metabolic strategies within a single habitat.

Microbial community composition at family level differed significantly between water column on the center of the lake and hot spring water (PERMANOVA, Bray–Curtis, *p* = 0.033, *R*^2^ = 0.62).

Similarly, the sediment at this location (HS12m) supports functions such as methanogenesis (favored by substrate availability and relatively high temperature), sulfate reduction, and potentially anaerobic ammonium oxidation (anammox). This convergence of overlapping biogeochemical cycles makes the zone at 12m from the spring a crucial hub of lake metabolism. Considering the significant contribution of hydrothermal discharge along the southeastern shore of the lake, the mixing of the two water masses could generate a dynamic front where intense elemental transformations occur, with potential impacts on the entire carbon cycle of the lake.

In conclusion, this study demonstrates that the pronounced physicochemical gradients in Lake Bagno dell'Acqua exert a major control on microbial community composition and functional potential. Clear ecological differentiation was observed between the alkaline lacustrine water mass and the hydrothermally influenced zones, each hosting distinct microbial assemblages. The transition interface where geothermal spring water mixes with the alkaline lake, was characterized by increased taxonomic richness, consistent with the coexistence of phototrophic, chemolithotrophic, and heterotrophic populations.

By integrating microbiological and geochemical analyses, this work highlights how extreme spatial heterogeneity and geothermal inputs contribute to the structuring of microbial niches in alkaline systems. These results provide new insights into microbial diversity patterns in such environments and represent the first detailed characterization of microbial communities in Lake Bagno dell'Acqua an ecosystem previously explored mainly for its geochemistry and astrobiological relevance.

## Data Availability

The datasets presented in this study can be found in online repositories. The data can be found here: https://www.ncbi.nlm.nih.gov/sra, PRJNA1039605.
