# Supplementary material for: Microbial community patterns in two geochemically contrasting zones within the alkaline lake Bagno dell'Acqua (Pantelleria Island, Italy)
Source: Front Microbiol. 2026 Feb 27;17:1773453. doi: 10.3389/fmicb.2026.1773453 (PMC12982464; doi:10.3389/fmicb.2026.1773453)
Supplement: Supplementary file 2 [file Supplementary_file_1.docx]

Supplementary Material

# Supplementary Figure


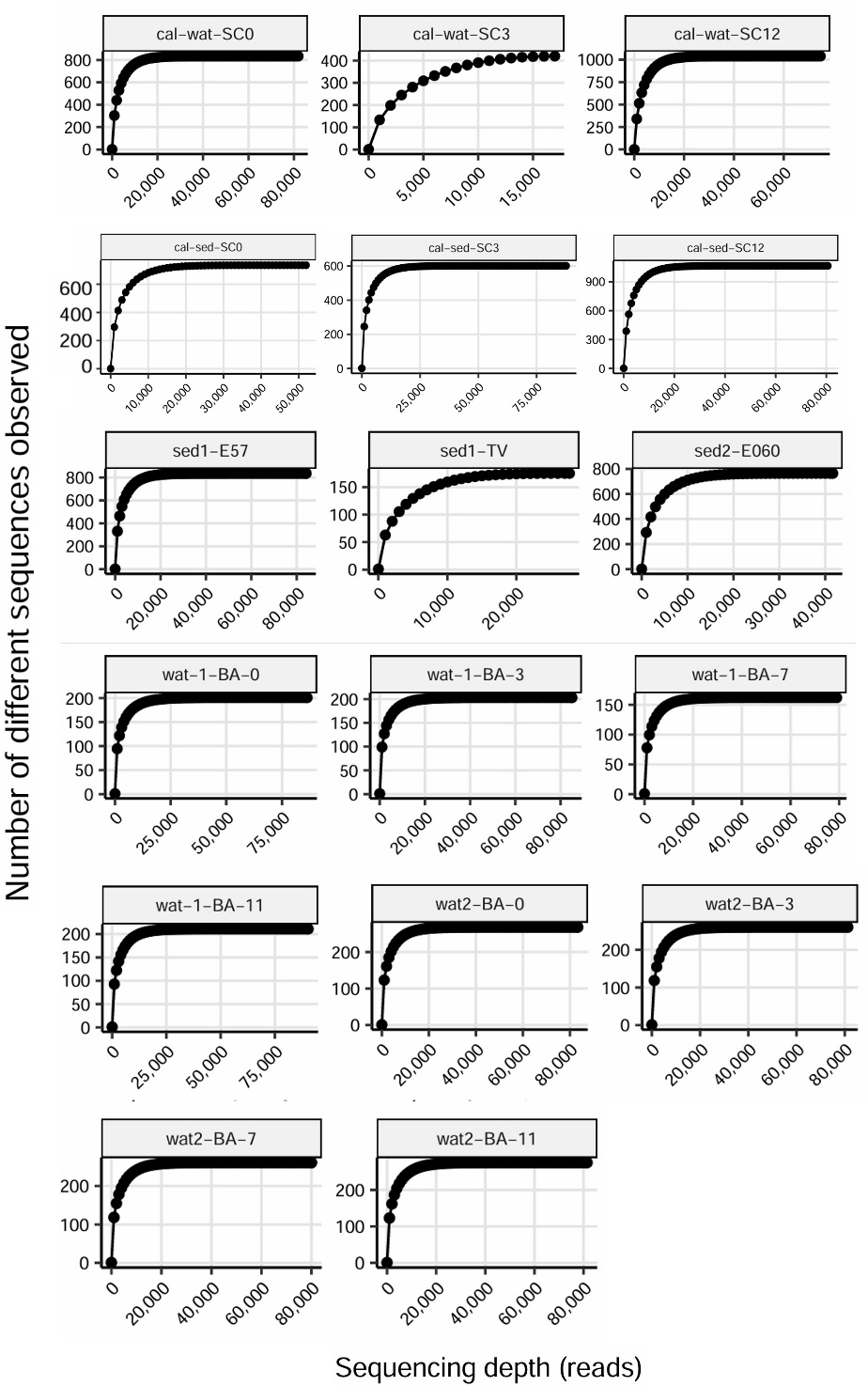


**Supplementary Figure 1.** Rarefaction curves for sequencing of samples. The x axis represents the number of sequences sampled whereas the y axis depicts the number of observed OTUs.
